# Leptospirosis and Respiratory Syncytial Virus (RSV) Co-infection: Diagnostic and Management Challenges

**DOI:** 10.7759/cureus.91428

**Published:** 2025-09-01

**Authors:** Kevin Koshy, Malyka Cheema

**Affiliations:** 1 Medicine, Royal Stoke Hospital, Stoke-On-Trent, GBR; 2 General Practice, Royal Stoke Hospital, Stoke-On-Trent, GBR

**Keywords:** antibiotic selection, leptospirosis, respiratory syncytial virus (rsv), tropical infections, weil's disease

## Abstract

Leptospirosis is one of the most widespread zoonotic infections worldwide, caused by spirochetes in the genus *Leptospira*. It is typically transmitted through exposure to contaminated water or soil from infected animals and is more common in tropical settings with frequent flooding. We discuss a 60-year-old gentleman with a background of malignant melanoma on immunotherapy who presented with a three-day history of fever, night sweats, and myalgia. He had recently returned from Tobago, where he had spent time swimming in the region's surrounding lakes, and also reported being bitten by mosquitoes and other insects. His observations and examination were unremarkable, with initial investigations, including routine blood tests, showing raised inflammatory markers and leukopenia; a chest X-ray revealed no acute findings. Following this, a throat swab was found to be positive for respiratory syncytial virus (RSV). Malaria, HIV, and hepatitis screenings were negative. His symptoms remained mild, and he was discharged after 24 hours with an outpatient follow-up. A rare and imported pathogens laboratory (RIPL) geographic panel was sent to identify other possible pathogens, given his travel history. The RIPL panel, which was reported six days later, was suggestive of acute leptospirosis (positive Lip16sDNA), and he was started on a two-week course of doxycycline. The non-specific nature of his symptoms highlights the importance of ensuring a thorough travel history is taken to identify leptospirosis in returning travelers. Furthermore, even if symptoms can easily be attributed to a more commonly seen infection such as RSV, this case highlights the need for maintaining a high index of suspicion for tropical diseases such as leptospirosis.

## Introduction

Leptospirosis is one of the most widespread zoonotic infections worldwide, caused by spirochetes in the genus *Leptospira* [1]. The infection is typically transmitted through exposure to soil and water contaminated by urine from infected animals such as rodents. It is more commonly observed in tropical settings, particularly where sanitation is inadequate and flooding is frequent. Symptoms are non-specific (including myalgia, fevers, and vomiting), making it hard to differentiate it from other infectious diseases. Although often self-limiting, some cases can lead to more severe complications, causing renal failure, jaundice, and aseptic meningitis. We discuss a patient who reported headaches, myalgia, fevers, and night sweats following a return from Tobago, who subsequently tested positive for respiratory syncytial virus (RSV). RSV could have solely been attributed to his presentation; however, given his travel history, further testing was performed, which identified a concurrent leptospirosis infection. We explore the challenges in diagnosing and managing patients with less severe clinical manifestations of leptospirosis.

This article was previously presented as a poster at the British Infection Association (BIA) 27th Annual Clinical and Scientific Meeting, including the Spring Trainee Day 2025, on 20 May 2025.

## Case presentation

A 60-year-old gentleman presented to the emergency department with a three-day history of fever, night sweats, generalized body aches, and a mild headache. He denied any shortness of breath or cough. His only significant medical history was malignant melanoma, for which he was on cycle six of immunotherapy (pembrolizumab). He had returned from a one-week holiday in Tobago five days prior to his attendance and reported having swum in the surrounding lakes multiple times during his stay. He also mentioned being bitten by mosquitoes and other insects. On examination, he appeared well, with observations within normal range and an unremarkable cardiorespiratory and abdominal examination.

Initial blood results showed mild leukopenia and lymphopenia, alongside an elevated C-reactive protein (CRP) level of 69 mg/L. A chest X-ray was also performed, which did not show any focal consolidation to suggest pneumonia as the underlying cause. A polymerase chain reaction (PCR) nasopharyngeal swab was also taken and found to be positive for RSV. He was admitted to the infectious diseases ward for further investigation and monitoring, given his presentation with a febrile illness on a background of foreign travel. Given the history of mosquito bites, malaria was initially considered the primary differential diagnosis; however, subsequent malaria screening returned a negative result. HIV, hepatitis, and blood cultures were also negative. A rare and imported pathogens laboratory (RIPL) geographical panel (serum samples) was also sent to identify other possible pathogens. While on the ward, two febrile episodes were noted, with all other observations remaining normal. Blood tests the following day also included liver function tests, which showed derangement more predominantly affecting the alanine transaminase (ALT) level compared to the alkaline phosphatase (ALP) level. Given there was no deterioration in his symptoms and his observations remained stable, he was subsequently discharged with outpatient follow-up under the infectious diseases team.

Six days post-discharge, the RIPL geographical panel had been completed, which was suggestive of acute leptospirosis (PCR testing positive for Lip16sDNA) while *Leptospira*-specific IgM serology was negative. Blood tests were also repeated, which showed an improvement in CRP (from 69 mg/L to 25 mg/L) and resolution of the leukopenia, lymphopenia, and liver function derangement observed prior to discharge (Table 1). He also reported a significant improvement in his symptoms, which correlated with his improved blood results. He was subsequently started on a two-week course of doxycycline, given his potential immunosuppression from his ongoing immunotherapy treatment.

**Table 1:** Blood results during admission and on follow-up * not measured

Results	Hemoglobin (135-180 g/L)	White cell count (4.0-11.0 x10^9^/L)	Neutrophils (2.0-7.0 x10^9^/L)	Lymphocytes (1.0-3.5 x10^9^/L)	Platelets (150-400 x10^9^/L)	Alanine transaminase (3-40 U/L)	Alkaline phosphatase (30-100 U/L)	Bilirubin (3-17 µmol/L)	C-reactive protein (<4 mg/L)	Creatinine (55-120 µmol/L)	Estimated glomerular filtration rate (>90 mL/minute/1.73 m^2^)
Day 1 (inpatient)	164	3.5	2.85	0.31	201	NM*	NM*	NM*	69	87	83
Day 2 (inpatient)	156	3.7	2.35	0.72	158	112	124	19	NM*	76	>90
Day 6 (follow-up)	142	6	2.78	2.01	506	49	94	11	25	71	>90

## Discussion

Leptospirosis typically presents with a febrile illness with associated symptoms such as myalgia, headaches, nausea, and a non-productive cough. The varied and often non-specific nature of symptoms can make it challenging to differentiate it from other tropical diseases such as malaria or dengue, as well as more ubiquitous viral illnesses such as RSV. This potential underdiagnosis of leptospirosis is supported by a study in Brazil, where further testing for leptospirosis was undertaken in patients presenting with febrile illnesses suspected to be due to dengue fever but who had tested negative for it [2]. It was found that among these 6,936 patients, 11.1% had antibodies against *Leptospira*, highlighting that even in a country with a high incidence rate of leptospirosis, the condition is not always clinically suspected, and other causes may be favored. Although the patient's reported generalized myalgia isn't specific to leptospirosis, the myalgia in leptospirosis often affects the lower back and calves in particular, which can be a useful distinguishing feature for clinicians to assess. Another differentiating factor, not observed in this case but one that occurs in a high proportion of patients, is conjunctival suffusion [3].

Furthermore, the positive finding of RSV in this case could easily have led to the diagnosis of leptospirosis being missed, with symptoms solely being attributed to the RSV infection. The combination of overlapping clinical features and the patient presenting during December (a prevalent time for RSV infections) further increased the risk of a missed diagnosis. This highlights the challenge of diagnosing leptospirosis and emphasizes the need for a high index of suspicion, especially in patients with epidemiological risk factors, such as travel to an endemic area during peak rainfall.

The variant of leptospirosis that presents with the self-limiting symptoms mentioned previously and which occurs in the majority of patients is known as anicteric leptospirosis. Some patients can then experience a subsequent immune phase of the illness, which can lead to uveitis and aseptic meningitis. However, in up to 10% of cases, symptoms can become more severe, leading to jaundice, renal failure, and pulmonary hemorrhage. This is known as icteric leptospirosis or Weil’s disease and is associated with a high mortality rate of up to 15% [4]. In this case, the patient had the anicteric variant, as no complications arose, and he was stable enough to be discharged. He was later notified of the RIPL panel findings. This was also reflected in his blood results, which showed improved inflammatory markers, renal function, and a transient rise in his liver function tests, indicating minimal hepatic involvement. However, it does highlight the importance of a timely follow-up in patients discharged with an undifferentiated febrile illness, as conditions such as leptospirosis can have a variable disease course and progression that may require further assessment.

The two main diagnostic tests used for leptospirosis are either PCR or serology testing for immunoglobulin (Ig) antibodies against *Leptospira*. PCR tests can be performed on either serum, urine, or cerebrospinal fluid samples. In this case, a RIPL panel was sent, which utilizes both serology and PCR testing to identify a wide range of pathogens based on the patient’s clinical presentation and travel history. The panel returned a positive PCR result, suggestive of leptospirosis, while the IgM test was negative. The negative IgM result is most likely due to the patient presenting too acutely for antibodies to be detected in the blood. This is why it is recommended that a second sample be taken one to two weeks after the first sample to determine if there is an increase in the titer [3]. This is supported by a study covering cases over a 10-year period, which showed that sensitivity rates for serology improved over time as the illness progressed from the acute to the convalescent stage [5]. On the other hand, PCR has proven to be a valuable diagnostic tool compared to serological methods in the initial stage of illness, with one study showing it identified up to 50% of cases that were negative with serology testing [6]. When suspecting leptospirosis, it is therefore crucial to perform the two diagnostic tests at the appropriate times (depending on the stage of illness) to minimize the risk of obtaining false-negative results.

Finally, there appears to be limited data on the benefits of antibiotics in leptospirosis, as symptoms often resolve in the majority of patients regardless of whether antibiotics are used. Doxycycline, which was the antibiotic of choice in this case, is the recommended treatment for mild cases of leptospirosis, with azithromycin and amoxicillin being suggested as alternatives [7]. A study conducted in Thailand found that both azithromycin and doxycycline are effective treatments for leptospirosis but noted that azithromycin is comparatively more expensive [8]. Other evidence supporting the use of antibiotics appears to be inconclusive, with studies citing insufficient data and failing to show a statistically significant benefit of antibiotics in reducing patient mortality [9,10]. However, the use of various classes of antibiotics has been found to reduce the defervescence time, which can help provide some symptom relief for the patient [11]. Although this patient’s symptoms were improving, his history of ongoing immunotherapy treatment with pembrolizumab was a contributing factor for antibiotics to be still initiated. Further studies investigating the course of leptospirosis, particularly in immunocompromised individuals, would be useful in guiding optimal management.

## Conclusions

This case emphasizes that investigating for tropical diseases, such as leptospirosis, should not be overlooked, even in the context of a patient having another confirmed infection, such as RSV. Although the symptoms were mild and did not progress in this case, there was a potential for the development of more severe symptoms and complications, which can be fatal. This reiterates the need to maintain a high index of suspicion in patients with clear risk factors that increase the likelihood of developing leptospirosis and subsequently arrange the appropriate testing and initiate treatment. Additional research into optimizing antibiotic strategies for leptospirosis, particularly in vulnerable patient groups such as those on immunotherapy, would also be valuable.
